# Syndrome de Fahr: une complication rare de la radiothérapie cérébrale survenant chez un patient acromégale

**DOI:** 10.11604/pamj.2014.18.287.5143

**Published:** 2014-08-12

**Authors:** Sahar El Aoud, Nadia Charfi

**Affiliations:** 1 Service d'Endocrinologie, CHU Hédi Chaker, sfax 3029, Tunisie

**Keywords:** Syndrome de Fahr, radiothérapie cérébrale, acromégalie, Fahr syndrome, brain radiotehrapy, acromegaly

## Image en medicine

Le Le syndrome de Fahr (SF) est caractérisé par la présence de calcifications intracérébrales, bilatérales et symétriques, des noyaux gris centraux. Il est souvent associé à des troubles du métabolisme phosphocalcique, et principalement à une hypoparathyroïdie. Il constitue une complication rare de la radiothérapie cérébrale probablement lié à une vascularite nécrotique. Nous rapportons l'observation d'un homme âgé de 27 ans qui était hospitalisé pour syndrome dysmorphique acrofacial. Le diagnostic d'une acromégalie secondaire à un macroadénome hypophysaire a été retenu. Une première exérèse fut réalisée, suivie d'une réintervention pour récidive et d'une radiothérapie complémentaire de 56 grays répartis sur 12 séances. L’évolution était marquée par la survenue d'une insuffisance antéhypophysaire. Cinq ans plus tard, le patient présentait des céphalées frontales. Un scanner cérébral objectivait un aspect stable de la tumeur, par ailleurs, il révélait des calcifications quasitotales des noyaux gris centraux, du tronc cérébral et du cervelet, de la substance blanche et de la jonction cortico-souscorticale absentes sur les examens neuroradiologiques précédents. Le diagnostic d'un syndrome de Fahr a été porté. Le bilan biologique ne révélait pas de troubles du métabolisme calcique, ni d'hypoparathyroidie. L'hypothyroidie et l'hypogonadisme ont été incriminés mais le caractère étendu des macrocalcifications n’était pas en faveur. Ainsi, l'origine post radique était retenue. Une surveillance clinique rapprochée était préconisée afin de déceler d’éventuelles complications neurologiques.

**Figure 1:**
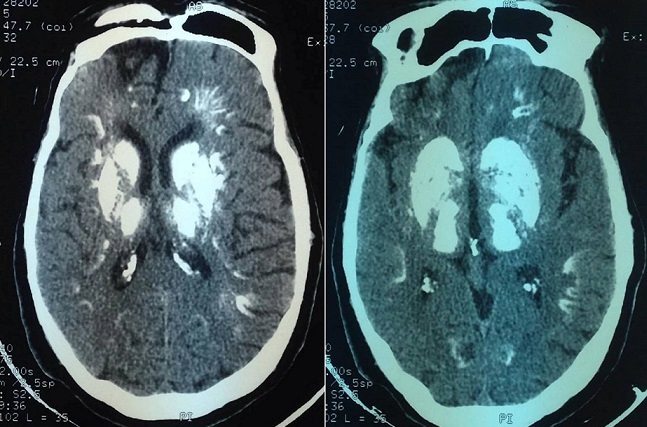
Syndrome de Fahr post-radique

